# A prediction model for moderate to severe acute kidney injury in people with heart failure

**DOI:** 10.1186/s40779-024-00558-z

**Published:** 2024-08-20

**Authors:** Yu-Qi Yang, Jing-Jing Da, Sheng Nie, Jing Yuan, Bi-Cheng Liu, Hua-Feng Liu, Qiong-Qiong Yang, Hua Li, Gang Xu, Jian-Ping Weng, Yao-Zhong Kong, Qi-Jun Wan, Gui-Sen Li, Chun-Bo Chen, Hong Xu, Ying Hu, Yong-Jun Shi, Yi-Lun Zhou, Guo-Bin Su, Ying Tang, Fan-Fan Hou, Yan Zha

**Affiliations:** 1 Department of Nephrology, Guizhou Provincial People’s Hospital, Guiyang, 550002 China; 2 National Clinical Research Center for Kidney Disease, State Laboratory of Organ Failure Research, Division of Nephrology, Nanfang Hospital, Southern Medical University, Guangzhou, 510515 China; 3 Institute of Nephrology, Zhongda Hospital, Southeast University School of Medicine, Nanjing, 210000 China; 4 Key Laboratory of Prevention and Management of Chronic Kidney Disease of Zhanjiang City, Institute of Nephrology, Affiliated Hospital of Guangdong Medical University, Zhanjiang, 524000 Guangdong China; 5 Department of Nephrology, Sun Yat-Sen Memorial Hospital, Sun Yat-Sen University, Guangzhou, 510515 China; 6 Sir Run Run Shaw Hospital, Zhejiang University School of Medicine, Hangzhou, 310000 China; 7 Division of Nephrology, Tongji Hospital, Tongji Medical College, Huazhong University of Science and Technology, Wuhan, 430000 China; 8 Department of Endocrinology, the First Affiliated Hospital of USTC, Division of Life Sciences and Medicine, University of Science and Technology of China, Hefei, 230000 China; 9 Department of Nephrology, the First People’s Hospital of Foshan, Foshan, 528000 Guangdong China; 10 The Second People’s Hospital of Shenzhen, Shenzhen University, Shenzhen, 518000 Guangdong China; 11 Department of Nephrology and Nephrology Institute, Sichuan Provincial People’s Hospital, School of Medicine, University of Electronic Science and Technology of China, Chengdu, 610072 China; 12 Department of Critical Care Medicine, Maoming People’s Hospital, Maoming, 525000 Guangdong China; 13 Children’s Hospital of Fudan University, Shanghai, 200000 China; 14 The Second Affiliated Hospital of Zhejiang University School of Medicine, Hangzhou, 310000 China; 15 Huizhou Municipal Central Hospital, Sun Yat-Sen University, Huizhou, 516000 Guangdong China; 16 Department of Nephrology, Beijing Tiantan Hospital, Capital Medical University, Beijing, 100000 China; 17 Department of Nephrology, Guangdong Provincial Hospital of Chinese Medicine, the Second Affiliated Hospital, the Second Clinical College, Guangzhou University of Chinese Medicine, Guangzhou, 510000 China; 18 The Third Affiliated Hospital of Southern Medical University, Guangzhou, 510000 China

**Keywords:** Acute kidney injury, Heart failure, Prediction model, Machine learning

Dear Editor,

Heart failure (HF) is a common multi-faceted and life-threatening syndrome, of which up to 23% occur acute kidney injury (AKI) [1]. HF-related AKI is largely overlooked or delayed in identification [2]. Approximately 85% of AKI cases that occurred during cardiac hospitalization in China were either ignored or identified too late [3]. Currently, there are no specific guidelines for the management of HF-related AKI. Hence, it is essential to identify patients at the risk of developing AKI and intervene promptly, to reduce social and economic burden.

Machine learning (ML)-based prediction models can predict patients at risk or likely to benefit from treatment, due to their superior capability in integrating data with multidimensional interactions. A variety of prediction models have been successfully used in HF patients, however, there remains a lack of widely accepted models for predicting HF-related AKI. Previous models have several critical limitations, including relatively low discrimination, miscellaneous patient selection, small sample sizes, and insufficient predictors [4, 5]. Hence, this study aimed to develop a prediction model of AKI in the HF population using ML algorithms. 

This study developed and validated a prediction model of moderate to severe AKI and AKI requiring dialysis, based on readily available variables from HF patients (Additional file 1: Table S1), using 4 ML algorithms with optimal hyperparameters (Fig. 1a; Additional file 1: Table S2). A total of 120,479 participants from the China Renal Data System (CDRS) database were included as the development cohort and 4327 patients from the local hospital as the external validation cohort. Additional file 1: Fig. S1 outlines the patient selection process for the development and validation cohorts. Additional file 1: Table S3 summarizes the baseline characteristics of the participants. In the development cohort [age 68.0 (58.0, 78.0) years; 71,895 men], 5975 (5.0%) patients developed moderate to severe AKI, and 2956 (2.5%) required dialysis. In the external validation cohort [age 72.0 (63.0, 79.0); 2722 men], patients were older, had worse baseline renal function, more severe HF conditions, and were less likely to take HF-associated medications, had a higher incidence of moderate to severe AKI (7.1%) and a lower risk of AKI requiring dialysis (1.1%), compared to the development cohort. Additional file 1: Table S4 demonstrate the comparison of baseline characteristics among patients in the three cohorts. Potential continuous variables with high multicollinearity and categorized variables with near-zero variance were removed (Additional file 1: Fig. S2).

**Fig. 1:**
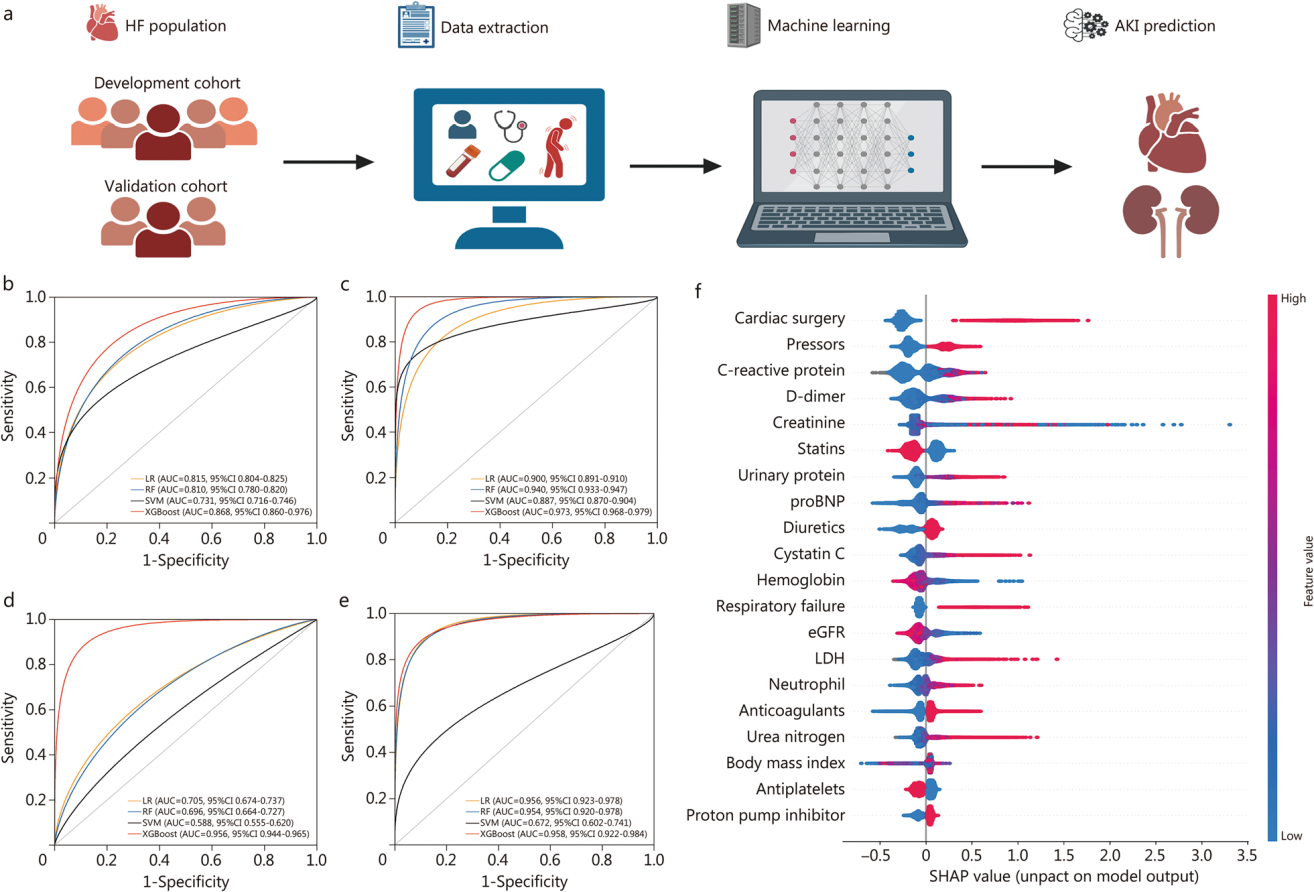
Development and validation of heart failure (HF)-related acute kidney injury (AKI) prediction model. **a** Study design. The area under the receiver operating characteristic curves (AUCs) for moderate to severe AKI (**b, d**) and AKI requiring dialysis (**c, e**) in the internal validation and external validation cohorts. **f** SHapley Additive exPlanation (SHAP) summary plot of the XGBoost model. The plot depicts the dot estimation on the model output of the XGBoost model. Each dot represents an individual patient from the dataset. Red represents the higher SHAP value of specific features; blue represents the lower SHAP value of specific features. The higher the SHAP values, the greater the risk of developing AKI development. LR logistic regression, RF random forest, SVM supported vector machine, XGBoost eXtreme gradient boosting, proBNP pro-brain natriuretic peptide, eGFR estimated glomerular filtration rate, LDH lactate dehydrogenase

The discrimination performance metrics of prediction models in the three cohorts are presented in Additional file 1: Table S5, Fig. S3, and Fig. 1b–e. Compared with the other three prediction models using logistic regression, supported vector machine and random forest, the eXtreme gradient boosting (XGBoost) model had the best predictive performance in discrimination with the highest area under the receiver operating characteristic curve (AUC) of 0.868 and 0.973; accuracy of 87.7% and 93.6%; sensitivity of 61.3% and 92.1%; specificity of 89.0% and 93.7%; positive predictive value (PPV) of 22.5% and 26.8%; negative predictive value (NPV) of 97.8% and 99.8%; for moderate to severe AKI (Fig. 1b) and AKI requiring dialysis (Fig. 1c) in the internal validation cohort; respectively. In the external validation cohort, the XGBoost model also attained the highest AUC of 0.956 and 0.958; accuracy of 95.7% and 99.1%; sensitivity of 43.8% and 30.4%; specificity of 99.6% and 99.8%; PPV of 90.0% and 60.9%; NPV of 95.9% and 99.3%; for moderate to severe AKI (Fig. 1d) and AKI requiring dialysis (Fig. 1e); respectively. These results demonstrated the trade-off of high NPV against low PPV, in order to minimize underreporting and heighten clinicians’ vigilance among high-risk patient groups, considering the life-threatening complications of HF-related AKI.

In addition, the XGBoost model was well-calibrated, as suggested by large calibration slopes (0.898, 1.044), small calibration intercept (0.026, −0.075), and small Brier’s score (0.039, 0.016) for moderate to severe AKI and AKI requiring dialysis in the internal validation cohort, respectively. Similar good calibrations with the calibration slopes of 1.740 and 1.039, the intercept of −0.096 and −0.138, and Brier’s scores of 0.038 and 0.012 were observed in the external validation cohort (Additional file 1: Table S6). It indicated that the model had preserved calibration.

To allow for the interpretation of our model’s predictions, we used SHapley Additive exPlanation values to assess feature importance and identified a feature’s relative contribution to uncover key features. Figure 1f shows the top 20 predictors in the XGBoost model, not only including renal health-related variables, but also several specific medication variables for the treatment of the primary causes of HF, HF symptoms, and HF-related complications, which might not be comparable to the general population.

In conclusion, this study developed, internally and externally validated a novel ML risk model for predicting HF-related AKI, based on a large dataset of Chinese admitted HF patients and uses of easily available variables. It is expected that the model could be integrated into the routine clinic workflow for risk stratification of the HF population and selecting individuals at high risk of AKI, facilitating early kidney-specific care, timely diagnosis, and treatment of HF-related AKI.

### Supplementary Information

**Additional file 1:** Materials and methods.** Table S1** List of 91 potential predictor variables used in the training models. **Table S2** Final hyperparameters adopted in the four ML models. **Table S3** Characteristics of the cohort participants in the prediction model of AKI outcomes. **Table S4** Characteristics of derivation, internal validation, and external validation cohorts according to AKI status. **Table S5** Discrimination performance of moderate to severe AKI, and AKI requiring dialysis risk prediction models for patients with HF in the derivation and validation cohorts. **Table S6** Calibration performance of various prediction models in the internal and external validation cohorts. **Fig. S1** Overview of study design. **Fig. S2** Multicollinearity results for continuous variables in the derivation cohorts. **Fig. S3** The AUCs for moderate to severe AKI and AKI requiring dialysis in the derivation cohorts.

## Data Availability

The data that support the findings of this study are available from the authors upon reasonable request.

## Abbreviations

AKI: Acute kidney injury
AUC: Area under the receiver operating characteristic curve
CRDS: China Renal Data System
HF: Heart failure
ML: Machine learning
NPV: Negative predictive value
PPV: Positive predictive value
XGBoost: EXtreme gradient boosting

## References

[1] Damman K, Valente MAE, Voors AA, O’Connor CM, van Veldhuisen DJ, Hillege HL. Renal impairment, worsening renal function, and outcome in patients with heart failure: an updated meta-analysis. Eur Heart J. 2014;35(7):455–69. 24164864 10.1093/eurheartj/eht386

[2] Ronco C, Bellomo R, Kellum JA. Acute kidney injury. Lancet. 2019;394(10212):1949–64. 31777389 10.1016/S0140-6736(19)32563-2

[3] Yang L, Xing G, Wang L, Wu Y, Li S, Xu G, et al. Acute kidney injury in China: a cross-sectional survey. Lancet. 2015;386(10002):1465–71. 26466051 10.1016/S0140-6736(15)00344-X

[4] Hong C, Sun Z, Hao Y, Dong Z, Gu Z, Huang Z. Identifying patients with heart failure who are susceptible to de novo acute kidney injury: machine learning approach. JMIR Med Inform. 2022;10(10):e37484. 36240002 10.2196/37484 PMC9617187

[5] Liu WT, Liu XQ, Jiang TT, Wang MY, Huang Y, Huang YL, et al. Using a machine learning model to predict the development of acute kidney injury in patients with heart failure. Front Cardiovasc Med. 2022;9:911987. 36176988 10.3389/fcvm.2022.911987 PMC9512707

