# Supplementary material for: A prediction model for moderate to severe acute kidney injury in people with heart failure
Source: Mil Med Res. 2024 Aug 20;11:57. doi: 10.1186/s40779-024-00558-z (PMC11334457; doi:10.1186/s40779-024-00558-z)
Supplement: Supplementary file 1 — Additional file 1: Materials and methods. Table S1 List of 91 potential predictor variables used in the training models. Table S2 Final hyperparameters adopted in the four ML models. Table S3 Characteristics of the cohort participants in the prediction model of AKI outcomes. Table S4 Characteristics of derivation, internal validation, and external validation cohorts according to AKI status. Table S5 Discrimination performance of moderate to severe AKI, and AKI requiring dialysis risk prediction models for patients with HF in the derivation and validation cohorts. Table S6 Calibration performance of various prediction models in the internal and external validation cohorts. Fig. S1 Overview of study design. Fig. S2 Multicollinearity results for continuous variables in the derivation cohorts. Fig. S3 The AUCs for moderate to severe AKI and AKI requiring dialysis in the derivation cohorts. [file 40779_2024_558_MOESM1_ESM.pdf]

Materials and methods

This study was approved by the Medical Ethics Committee of Nanfang Hospital, Southern Medical University (NFEC-2019-213), and the Medical Ethics Committee of Guizhou Provincial People's Hospital ([2019]29). The study followed Transparent Reporting of a Multivariable Prediction Model for Individual Prognosis or Diagnosis (TRIPOD) guidance for prediction model development and validation. The study was conducted in compliance with the principles of the Declaration of Helsinki and its later revisions.

Study population

Development cohort

Participants were recruited from the China Renal Data System (CRDS), which was a large and representative electronic medical database in China managed by the National Clinical Research Center for Kidney Disease and the China Center for Disease Control and Prevention. The database contains more than 7 million inpatients and outpatients from 19 large, urban academic centers that cover the major geographic regions across China from 1 January 2000 to 26 May 2021. Each participating center exported both the inpatient and outpatient follow-up data for each patient from its proprietary hospital information systems, including demographic information, details of prescriptions, the diagnosis, the results of laboratory testing, information regarding surgery, and vital signs. The exported data were cleaned up, standardized, anonymized, and pooled at the CRDS data center. All the laboratories at the participating hospitals had passed the annual External Quality Assessment of the Chinese National Center for Clinical Laboratories. CRDS database has achieved the ISO9001 quality certification and the approval of the China Office of Human Genetic Resources for Data Preservation Application from the Ministry of Science and Technology of China (approval No. 2021-BC0037). For data cleaning and standardization, we conducted two-step quality control for the raw data and the standardized data to ensure the high quality of our database. First, a portion of the raw data from each hospital was randomly extracted and reviewed to verify the accuracy and completeness of at least 95%. Second, quality control was implemented as feedback on the data cleaning cycles, by clinical staff manually reviewing the data and submitting the QC report. The data cleaning process would continuously cycle until the quality control accuracy reached 95%. In this study, 120,479 HF participants from the CDRS database were finally included in the development cohort. A split-sample approach was used to develop and internally validate the prediction model, with a random sample of 70% used for the derivation cohort, and the remaining 30% used for the internal validation cohort (**Additional file 1: Fig. S1**).

External validation cohort

To ensure the generalizability of the derived model for prospective use, we validated our results in an external

validation cohort from Guizhou Provincial People's Hospital, a sub-unit hospital of CDRS with a relatively complete clinical record. The validation cohort comprised 4327 adult HF patients between 1 January 2010 and 30 May 2022, by implementing the same inclusion and exclusion criteria, without overlap with the development cohort.

Participants

Patients in both cohorts were eligible if they were aged ≥ 18 years and admitted with HF within the inclusion time frame. The diagnosis of HF was based on the International Classification of Diseases, 10th Revision (ICD-10) code I50. Only the first admission record was used in patients with multiple hospital admissions. The exclusion criteria were as follows: 1) patients who had end-stage renal disease [defined as estimated glomerular filtration rate (eGFR) < 15 ml/(min·1.73 m^2)], maintenance dialysis, or kidney transplantation prior to admission; 2) patients who had heart transplantation prior to admission; 3) patients who have nephrectomy prior to admission; 4) patients who have been diagnosed urinary obstruction, kidney trauma, sepsis, or malignant tumor within one month; 5) patients who had pregnancy at the time of this admission; and 6) patients who missed serum creatinine (SCr) data for two times within 7 d during this admission. All participants were categorized into three groups according to the cohorts: derivation cohort, internal validation cohort, and external validation cohort. Participants in each cohort were then categorized into two groups according to the status of kidney function: non-moderate to severe AKI group and moderate to severe AKI group.

Study outcomes

AKI was defined as an increase in SCr by 0.3 mg/dl within 48 h or a 50% increase in SCr from the baseline within 7 d according to the Kidney Disease Improving Global Outcomes (KDIGO) guidelines [1]. Urine output criteria were not used because urine volume was not available in this study. As the previously described algorithm [2], the SCr data within 7 d prior to the admission were sorted in increasing order according to the test time. At any time point "t", a baseline SCr was dynamically defined as the mean of SCr levels within the 7 d before "t", and each of the available SCr data within 7 d after "t" was compared with this baseline. AKI was labeled only once for admission. The earliest day that the SCr change met the KDIGO criteria was defined as the date of AKI onset. AKI stages were determined by the peak SCr level after AKI detection or initiation of dialysis, with a rise of less than 100% indicating stage 1, a rise of 100% or more indicating stage 2, and a rise of 200% or more over baseline or initiation of dialysis indicating stage 3.

The primary outcome was moderate to severe AKI, defined as AKI stage 2 or stage 3. The secondary outcome was AKI requiring dialysis, defined as AKI who initiated dialysis treatment.

Predictors

In the CRDS, both categorical and continuous data were obtained as predictors. A total of 91 candidate features were identified, including 2 demographic information (age and sex), 6 vital traits, 23 comorbidities, 20 medications, 1 operation, and 39 laboratory characteristics (**Additional file 1: Table S1**). Vital traits were extracted at the time of this admission. Comorbidities were identified by ICD-10 codes prior to admission. Medications prescribed within 3 months prior to admission were classified according to the Anatomical Therapeutic Chemical classification system and confirmed by drug name. Operations prior to admission were identified by the ICD-9 operation classification system. These categorical features were recorded as binary indicators, and zero or near-zero variance features (binary variables with ≤ 0.1% prevalence in the derivation cohort) were removed. Baseline laboratory characteristics were extracted as the latest value prior to admission or the first after admission, whichever occurred earlier. Multicollinearity was assessed among the predictor variables using correlation coefficients and variance inflation factor. For paired continuous variables with correlation coefficients > 0.8 or variance inflation factor > 10, we removed the variables that had higher correlation coefficients with other variables. The variables with missing data more than 25% were excluded. For algorithms that cannot handle missing predictor data, multiple imputations were performed for missing values using multiple imputations based on the random forest method. The imputation models included the primary outcome and any baseline variable without missing data.

Development of prediction models

Four models were trained to predict the diagnostic outcome of AKI using the full available features in the derivation cohort: 1) stepwise logistical regression (LR); 2) supported vector machine (SVM); 3) random forest (RF); and 4) eXtreme gradient boosting (XGBoost). In the conventional statistical model, all potential predictors were included at the start, followed by a stepwise selection by LR which added and dropped predictors to identify a model with the lowest Akaike Information Criteria [3], penalizing adding variables into the model. For three ML algorithms, the 10-fold cross-validation was used to evaluate the performance of each model, avoid any overfitting/underfitting, ensure the robustness of models, and minimize bias. Specifically, an inner tenfold cross-validation was applied to tune the hyperparameters with a random gird search, to maximize the area under the receiver operating characteristic curve (AUC). The final hyperparameters used in the prediction models are listed in **Additional file 1: Table S2**.

To allow for the interpretation of our models' predictions, we assessed feature importance and identified a feature's relative contribution to uncover key features using the SHapley Additive exPlanation (SHAP) values, which is a unified approach for explaining the outcome of any ML model and provide consistent and

locally accurate attribution values for each feature within each prediction model [4].

Evaluation of prediction models

We reported the model performance in terms of discrimination and calibration. For the overall discriminatory ability of models, we reported standard diagnostic accuracy estimates, AUC along with 95% confidence interval (CI), sensitivity, specificity, positive predictive value (PPV), negative predictive value (NPV), accuracy along with 95%CI, precision, recall, and F1 score measures, to evaluate the performance of models in the presence of class imbalance in the derivation, internal and external validation cohorts. The optimal cut-off value for risk classification was determined based on the AUC analysis of the training dataset using Youden's index.

As a measure of both discrimination and calibration, the Brier score was calculated as the mean square error between the actual event and estimated probability [5]. The calibration slope and intercept were evaluated [6]. A smaller Brier score, a calibration slope closer to 1, and an intercept closer to 0 imply better calibration.

Statistical analysis

All analyses were conducted in R (version 4.3.1; R Project for Statistical Computing, https://www.r-project.org), and Python (version 3.9.3; Python Software Foundation, http://www.python.org).

Descriptive statistics are presented as M (Q_1, Q_3) for continuous variables and n (%) for categorical variables. The differences among different groups were compared using χ^2 tests for categorical variables, and Wilcoxon rank-sum for continuous groups. All continuous variables included in the models were fitted in restricted cubic splines with 3 knots based on covariate distributions (at the 10th, 50th, and 90th percentiles) to allow for flexible nonlinear associations with the primary outcome measures using multivariable LR. A significant level was set at $P < 0.05$.

Table S1 List of 91 potential predictor variables used in the training models

**Predictor variables**	
Demographics	
1	Age
2	Sex
Examinations	
3	Body mass index (BMI)
4	Systolic blood pressure (SBP)
5	Diastolic blood pressure (DBP)
6	Pulse
7	Respiratory rate
8	Heart rate (HR)
Diagnosis	
9	Diabetes mellitus
10	Hypertension
11	Chronic obstructive pulmonary diseases
12	Chronic pulmonary diseases
13	Asthma
14	Pulmonary heart diseases
15	Respiratory failure
16	Stroke
17	Cardiovascular diseases
18	Myocardial infarction
19	Congestive heart diseases
20	Valvular heart diseases
21	Conductive diseases
22	Arrhythmia
23	Cerebrovascular diseases
24	Liver diseases
25	Liver cirrhosis
26	Glomerular diseases
27	Chronic kidney diseases
28	Connective tissue diseases
29	Peripheral vascular diseases
30	Dyslipidemia
31	Hyperuricemia
Operations	
32	Cardiac surgery
Medications	
33	Proton pump inhibitors
34	Diuretics
35	Statins
36	Antiplatelets
37	Anticoagulants
38	Pressors
39	Renin angiotensinogen aldosterone inhibitors
40	Calcium channel blockers
41	β blockers

42	Inotropes
43	Antiarrhythmic agents
44	Nitrates
45	Corticoids
46	Antibiotics
47	Antifungals
48	Antivirals
49	Antidiabetic agents
50	Contrasts
51	Antilipidemic agents
52	Non-steroidal anti-inflammatory drugs (NSAIDs)
Laboratory variables	
53	Blood urea nitrogen (BUN)
54	Cardiac troponin I (cTNI)
55	Cystatin C (CyscC)
56	Direct bilirubin (DBIL)
57	D-dimer
58	Fibrinogen (FIB)
59	Gamma-glutamyltransferase (GGT)
60	Glucose
61	Neutrophil
62	Hemoglobin (Hb)
63	Homocysteine (Hcy)
64	High-density lipoprotein
65	Indirect bilirubin (IBIL)
66	International normalized ratio (INR)
67	Lactate dehydrogenase (LDH)
68	Low-density lipoprotein
69	Lymphocyte (Lymph)
70	Sodium
71	Phosphorus
72	Platelet count (PLT)
73	Prothrombin time (PT)
74	Total bilirubin (TBIL)
75	Red blood count (RBC)
76	Total bile acid (TBA)
77	Total cholesterol (TCHO)
78	Triglyceride (TG)
79	Total protein (TP)
80	Uric acid (UA)
81	Urine protein (PRO)
82	White blood count (WBC)
83	Estimated glomerular filtration rate (eGFR)
84	Pro-brain natriuretic peptide (proBNP)
85	Alkaline phosphatase (ALP)
86	Albumin (ALB)
87	Alanine aminotransferase (ALT)
88	Aspartate aminotransferase (AST)
89	Activated partial thromboplastin time (APTT)

90	Calcium
91	C-reactive protein (CRP)

Table S2 Final hyperparameters adopted in the four ML models

**ML models**	**Hyperparameters**
SVM	kernel = "radial"<br>gamma = 0.05<br>cost = 100
RF	m_{try} = 9<br>n_{tree} = 873
XGBoost	learning_rate = 0.05<br>n_estimators = 2000<br>max_depth = 8<br>gamma = 0.2<br>colsample_bytree = 0.4<br>min_child_weight = 5<br>subsample = 1.0
LR	-

ML machine learning, *SVM* supported vector machine, *RF* random forest, *XGBoost* eXtreme gradient boosting, *gamma* minimum loss reduction, *LR* logistic regression m_{try} number of randomly selected predictors, n_{tree} number of boosting iterations, *max_depth* maximum tree depth, *colsample_bytree* subsample ratio of columns, *min_child_weight* minimum sum of instance weight, *subsample* subsample percentage, "-" indicates no data

Table S3 Characteristics of the cohort participants in the prediction model of AKI outcomes

**Characteristics**	**Development cohort**		**External validation cohort (n = 4327)**
	**Derivation cohort (n = 84,335)**	**Internal validation cohort (n = 36,144)**	
Moderate to severe AKI [n(%)]	4182 (5.0)	1793 (5.0)	308 (7.1)*
AKI requiring dialysis [n(%)]	2065 (2.4)	891 (2.5)	46 (1.1)*
Age [years, M (Q_1, Q_3)]	68.0 (58.0, 78.0)	68.0 (58.0, 78.0)	72.0 (63.0, 79.0)*
Sex [n(%)]			
Female	34,004 (40.3)	14,580 (40.3)	1605 (37.1)*
Male	50,331 (59.7)	21,564 (59.7)	2722 (62.9)*
BMI [kg/m^2, M (Q_1, Q_3)]	23.4 (20.8, 26.0)	23.4 (20.8, 26.0)	23.0 (20.4, 24.5)*
Medication [n(%)]			
Diuretics	51,447 (61.0)	22,054 (61.0)	1606 (37.1)*
Statins	46,775 (55.5)	20,231 (56.0)	1234 (28.5)*
Antiplatelets	44,129 (52.3)	18,988 (52.5)	1239 (28.6)*
Anticoagulants	40,728 (48.3)	17,517 (48.5)	801 (18.5)*
Pressors	12,663 (15.0)	5477 (15.2)	404 (9.3)*
Proton pump inhibitors	46,221 (54.8)	19,783 (54.7)	1209 (27.9)*
Comorbidity [n(%)]			
Respiratory failure	5359 (6.4)	2236 (6.2)	1112 (25.7)*
Cardiac surgery	11,721 (13.9)	5077 (14.0)	42 (1.0)*
Laboratory variables [M (Q_1, Q_3)]			
Hb (g/L)	128.0 (111.6, 142.0)	128.0 (112.0, 142.0)	132.0 (115.0, 147.0)*
Neutrophil (×10^9/L)	5.0 (3.6, 7.3)	5.0 (3.6, 7.4)	5.0 (3.6, 7.1)
proBNP (pg/ml)	1456 (318, 4360)	1446 (319, 4388)	2142 (562, 5573)*
BUN (mmol/L)	6.3 (4.8, 8.9)	6.3 (4.8, 8.8)	6.8 (5.0, 9.6)*
SCr (μmol/L)	80.5 (64.8, 105.0)	80.3 (64.8, 104.3)	84.7 (68.4, 110.0)*
eGFR [ml/(min·1.73 m^2)]	78.5 (55.6, 93.5)	78.6 (55.8, 93.5)	72.3 (52.6, 88.8)*
CyscC (mg/L)	1.1 (0.9, 1.6)	1.1 (0.9, 1.5)	1.3 (1.1, 1.8)*
D-dimer (μg/ml)	0.7 (0.3, 1.8)	0.7 (0.3, 1.7)	1.6 (0.8, 3.5)*
LDH (U/L)	217.0 (175.1, 291.6)	216.4 (176.0, 291.0)	251.0 (202.0, 329.0)*
CRP (mg/L)	5.0 (2.0, 18.3)	5.0 (2.0, 18.4)	11.8 (3.20, 46.8)*

*P<0.05, external validation cohort vs. derivation cohort. *AKI* acute kidney injury, *Hb* hemoglobin, *proBNP* pro-brain natriuretic peptide, *BUN* blood urea nitrogen, *SCr* serum creatinine, *eGFR* estimated glomerular filtration rate, *CyscC* cystatin C, *LDH* lactate dehydrogenase, *CRP* C-reactive protein

Table S4 Characteristics of derivation, internal validation and external validation cohorts according to AKI status

Characteristics	Derivation cohort		Internal validation cohort		External validation cohort	
	**Non-moderate to severe AKI (*n* = 80,153)**	**Moderate to severe AKI (*n* = 4182)**	**Non-moderate to severe AKI (*n* = 34,351)**	**Moderate to severe AKI (*n* = 1793)**	**Non-moderate to severe AKI (*n* = 4019)**	**Moderate to severe AKI (*n* = 308)**
Age [years, M (Q_1, Q_3)]	68.0 (58.0, 78.0)	68.0 (55.0, 78.0)	68.0 (58.0, 78.0)	68.0 (56.0, 78.0)	72.0 (63.0, 79.0)	73.5 (59.0, 79.0)
Sex [*n*(%)]						
Female	32,139 (40.1)	1865 (44.6)	13,787 (40.1)	793 (44.2)	1500 (37.3)	105 (34.1)
Male	48,014 (59.9)	2317 (55.4)	20,564 (59.9)	1000 (55.8)	2519 (62.7)	203 (65.9)
BMI [kg/m^2, M (Q_1, Q_3)]	23.4 (20.9, 26.0)	22.8 (20.0, 25.5)	23.4 (20.9, 26.0)	22.8 (19.8, 25.4)	23.7 (21.2, 26.7)	22.0 (18.7, 24.6)
Medication [*n* (%)]						
Diuretics	48,100 (60.0)	3347 (80.0)	20,625 (60.0)	1429 (79.7)	1473 (36.7)	133 (43.2)
Statins	45,249 (56.5)	1526 (36.5)	19,533 (56.9)	678 (37.8)	1156 (28.8)	78 (25.3)
Antiplatelets	42,721 (53.3)	1408 (33.7)	18,377 (53.5)	611 (34.1)	1159 (28.8)	80 (26.0)
Anticoagulants	38,205 (47.7)	2523 (60.3)	16,433 (47.8)	1084 (60.5)	730 (18.2)	71 (23.1)
Pressors	11,225 (14.0)	1438 (34.4)	4876 (14.2)	601 (33.5)	332 (8.3)	72 (23.4)
Proton pump inhibitors	43,477 (54.2)	2744 (65.6)	18,635 (54.2)	1148 (64.0)	1077 (26.8)	132 (42.9)
Comorbidity [*n* (%)]						
Respiratory failure	4620 (5.8)	739 (17.7)	1953 (5.7)	283 (15.8)	990 (24.6)	122 (39.6)
Cardiac surgery	10,735 (13.4)	986 (23.6)	4633 (13.5)	444 (24.8)	33 (0.8)	9 (2.9)
Laboratory variables [M (Q_1, Q_3)]						
Hb (g/L)	128.0 (113.0, 142.0)	112.0 (87.0, 132.0)	129.0 (113.0, 142.0)	113.0 (89.0, 132.4)	132.0 (116.0, 147.0)	129.4 (105.0, 148.3)
Neutrophil (×10^9/L)	4.96 (3.60, 7.25)	6.26 (3.97, 9.91)	4.97 (3.60, 7.30)	6.04 (4.04, 9.61)	4.9 (3.5, 7.1)	5.3 (4.0, 8.2)
proBNP (pg/ml)	1372.0 (297.0, 4077.0)	4654.0 (1352.5, 15,000.0)	1357.0 (297.0, 4108.5)	4778.0 (1398.0, 14,894.0)	2046.0 (548.3, 5456.0)	2878.0 (764.7, 7856.5)
BUN (mmol/L)	6.3 (4.8, 8.6)	9.2 (6.0, 17.7)	6.2 (4.8, 8.6)	9.0 (5.9, 17.8)	6.7 (5.0, 9.3)	8.7 (5.9, 14.4)
SCr (μmol/L)	80.0 (64.6, 103.0)	105.5 (69.4, 233.0)	79.8 (64.6, 102.4)	105.0 (71.0, 240.0)	83.9 (68.1, 107.0)	102.4 (73.4, 173.9)
eGFR [ml/(min·1.73 m^2)]	79.1 (57.1, 93.7)	54.0 (21.0, 87.1)	79.2 (57.2, 93.7)	54.9 (20.5, 87.1)	73.4 (53.7, 88.9)	56.4 (30.1, 85.1)
CyscC (mg/L)	1.1 (0.9, 1.5)	1.6 (1.1, 3.2)	1.1 (0.9, 1.5)	1.7 (1.1, 3.1)	1.3 (1.1, 1.7)	1.6 (1.2, 2.4)
D-dimer (μg/ml)	0.7 (0.3, 1.7)	1.7 (0.7, 3.8)	0.7 (0.3, 1.7)	1.6 (0.6, 3.9)	1.6 (0.8, 3.4)	2.3 (1.2, 5.7)
LDH (U/L)	214.6 (174.4, 286.5)	271.0 (207.0, 410.0)	214.0 (174.9, 286.0)	268.7 (207.0, 395.1)	249.0 (199.0, 326.0)	275.0 (217.0, 388.8)
CRP (mg/L)	5.0 (1.9, 17.3)	11.6 (4.7, 51.3)	5.0 (1.9, 17.3)	11.1 (4.3, 53.7)	11.4 (3.0, 45.6)	19.5 (6.0, 76.6)

AKI acute kidney injury, *BMI* body mass index, *Hb* hemoglobin, *proBNP* pro-brain natriuretic peptide, *BUN* blood urea nitrogen, *SCr* serum creatinine, *eGFR* estimated glomerular filtration rate, *CyscC* cystatin C, *LDH* lactate dehydrogenase, *CRP* C-reactive protein

Table S5 Discrimination performance of moderate to severe AKI, and AKI requiring dialysis risk prediction models for patients with HF in the derivation and validation cohorts

Algorithm used in model development		AUC (95%CI)	Accuracy (%)	Sensitivity (%)	Specificity (%)	PPV (%)	NPV (%)	F1 score	Recall (%)
Derivation cohort									
Moderate to severe AKI	LR	0.821 (0.815 – 0.827)	69.2 (68.9 – 69.6)	80.1	68.7	11.8	98.5	0.205	80.1
	SVM	0.929 (0.923 – 0.934)	97.5 (97.4 – 97.6)	82.0	98.3	71.7	99.0	0.765	82.0
	RF	1.000	100.0	100.0	100.0	100.0	100.0	1.000	100.0
	XGBoost	0.949 (0.946 – 0.952)	90.0 (89.8 – 90.2)	84.4	90.3	31.3	99.1	0.457	84.4
AKI requiring dialysis	LR	0.899 (0.893 – 0.906)	79.2 (79.0 – 79.5)	84.2	79.1	9.2	99.5	0.167	84.2
	SVM	0.891 (0.880 – 0.901)	93.3 (93.1 – 93.5)	81.4	93.6	24.2	99.5	0.374	81.4
	RF	0.973 (0.970 – 0.976)	93.4 (93.3 – 93.6)	92.0	93.5	26.1	92.0	0.407	92.0
	XGBoost	0.991 (0.990 – 0.993)	95.5 (95.3 – 95.6)	95.4	95.5	34.8	99.9	0.510	95.4
Internal validation cohort									
Moderate to severe AKI	LR	0.815 (0.804 – 0.825)	69.6 (69.1 – 70.1)	78.1	69.2	11.7	98.4	0.203	78.1
	SVM	0.731 (0.716 – 0.746)	85.9 (85.5 – 86.2)	51.5	87.7	17.9	97.2	0.266	51.5
	RF	0.810 (0.800 – 0.820)	95.1 (94.9 – 95.3)	9.2	99.6	53.5	95.6	0.157	9.2
	XGBoost	0.868 (0.860 – 0.976)	87.7 (87.3 – 88.0)	61.3	89.0	22.5	97.8	0.329	61.4
AKI requiring dialysis	LR	0.900 (0.891 – 0.910)	79.3 (78.8 – 79.7)	84.0	79.1	9.2	99.5	0.166	84.0
	SVM	0.887 (0.870 – 0.904)	93.4 (93.1 – 93.6)	81.9	93.6	24.5	99.5	0.377	82.0
	RF	0.940 (0.933 – 0.947)	94.0 (93.7 – 94.2)	70.3	94.6	24.2	99.2	0.360	70.3
	XGBoost	0.973 (0.968 – 0.979)	93.6 (93.4 – 93.9)	92.1	93.7	26.8	99.8	0.415	92.1
External validation cohort									
Moderate to severe AKI	LR	0.705 (0.674 – 0.737)	71.1 (69.7 – 72.4)	58.1	72.1	13.8	95.7	0.223	58.1
	SVM	0.588 (0.555 – 0.620)	92.8 (92.0 – 93.6)	0.3	99.9	25.0	92.9	0.006	0.3
	RF	0.696 (0.664 – 0.727)	92.5 (91.7 – 93.3)	6.5	99.1	36.4	93.3	0.110	6.5
	XGBoost	0.956 (0.944 – 0.965)	95.7 (95.0 – 96.2)	43.8	99.6	90.0	95.9	0.590	43.8
AKI requiring dialysis	LR	0.956 (0.923 – 0.978)	81.3 (80.1 – 82.4)	95.7	81.1	5.2	99.9	0.098	95.7
	SVM	0.672 (0.602 – 0.741)	98.8 (98.5 – 99.1)	44.4	99.3	34.8	99.5	0.390	34.8
	RF	0.954 (0.920 – 0.978)	90.3 (89.4 – 91.1)	82.6	90.4	8.4	99.8	0.153	8.4
	XGBoost	0.958 (0.922 – 0.984)	99.1 (98.7 – 99.3)	30.4	99.8	60.9	99.3	0.406	30.4

HF heart failure, *LR* logistic regression, *SVM* supported vector machine, *RF* random forest, *XGBoost* eXtreme gradient boosting, *AUC* area under the receiver operating characteristic curve, *CI* confidence interval, *PPV* positive predictive value, *NPV* negative predictive value, *AKI* acute kidney injury

Table S6 Calibration performance of various prediction models in the internal and external validation cohorts

Algorithm used in model development	Brier's score	Calibration	
		Slope	Intercept
Internal validation cohort			
Moderate to severe AKI			
LR	0.042	0.579	0.089
SVM	0.043	0.566	0.177
RF	0.042	1.170	-0.056
XGBoost	0.039	0.898	0.026
AKI requiring dialysis			
LR	0.021	0.686	0.027
SVM	0.019	0.574	0.225
RF	0.018	0.845	0.045
XGBoost	0.016	1.044	-0.075
External validation cohort			
Moderate to severe AKI			
LR	0.066	0.316	0.107
SVM	0.070	2.402	-0.115
RF	0.064	0.594	0.042
XGBoost	0.038	1.740	-0.096
AKI requiring dialysis			
LR	0.011	0.551	0.003
SVM	0.011	0.144	-0.063
RF	0.009	0.616	0.069
XGBoost	0.012	1.039	-0.138

AKI acute kidney injury, *LR* logistic regression, *SVM* supported vector machine, *RF* random forest, *XGBoost* eXtreme gradient boosting

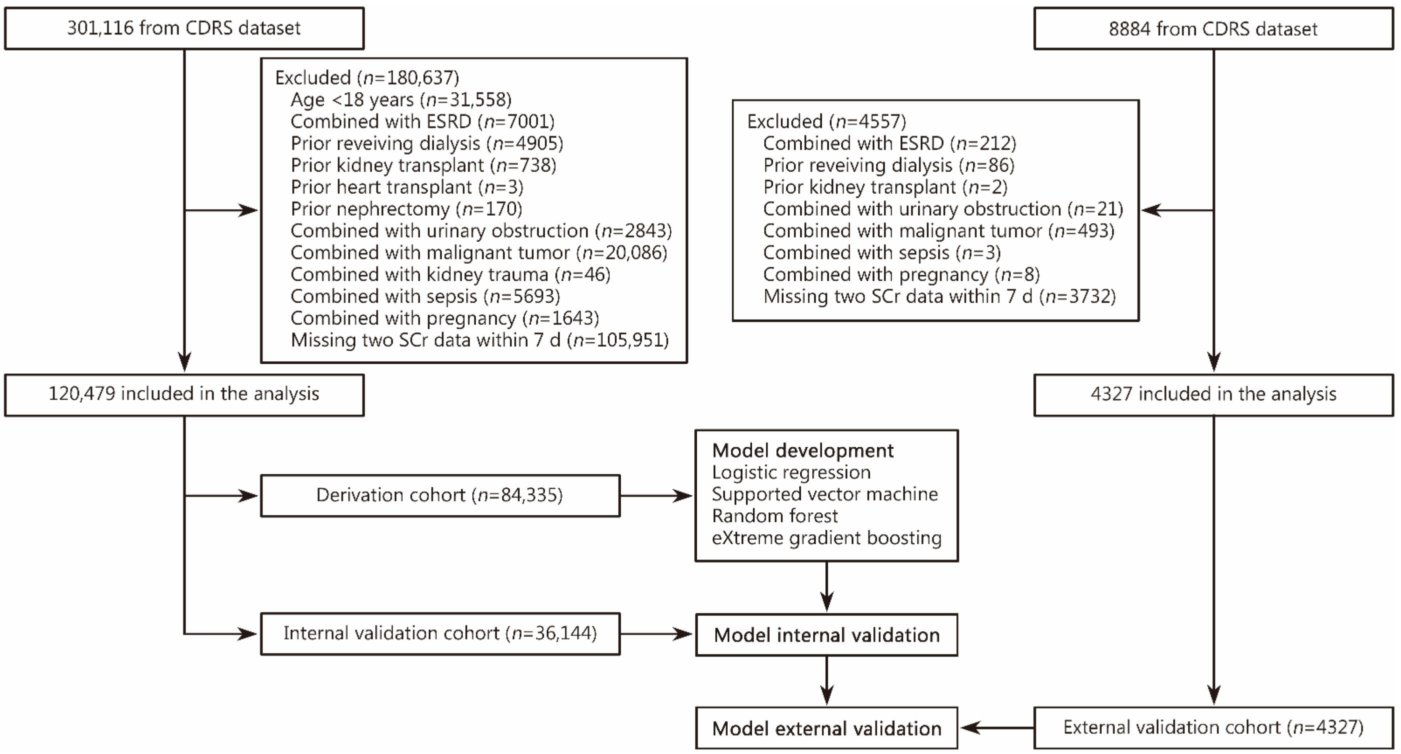

Fig. S1 Overview of study design. CDRS China Renal Data System, ESRD end-stage renal disease, SCr serum creatinine

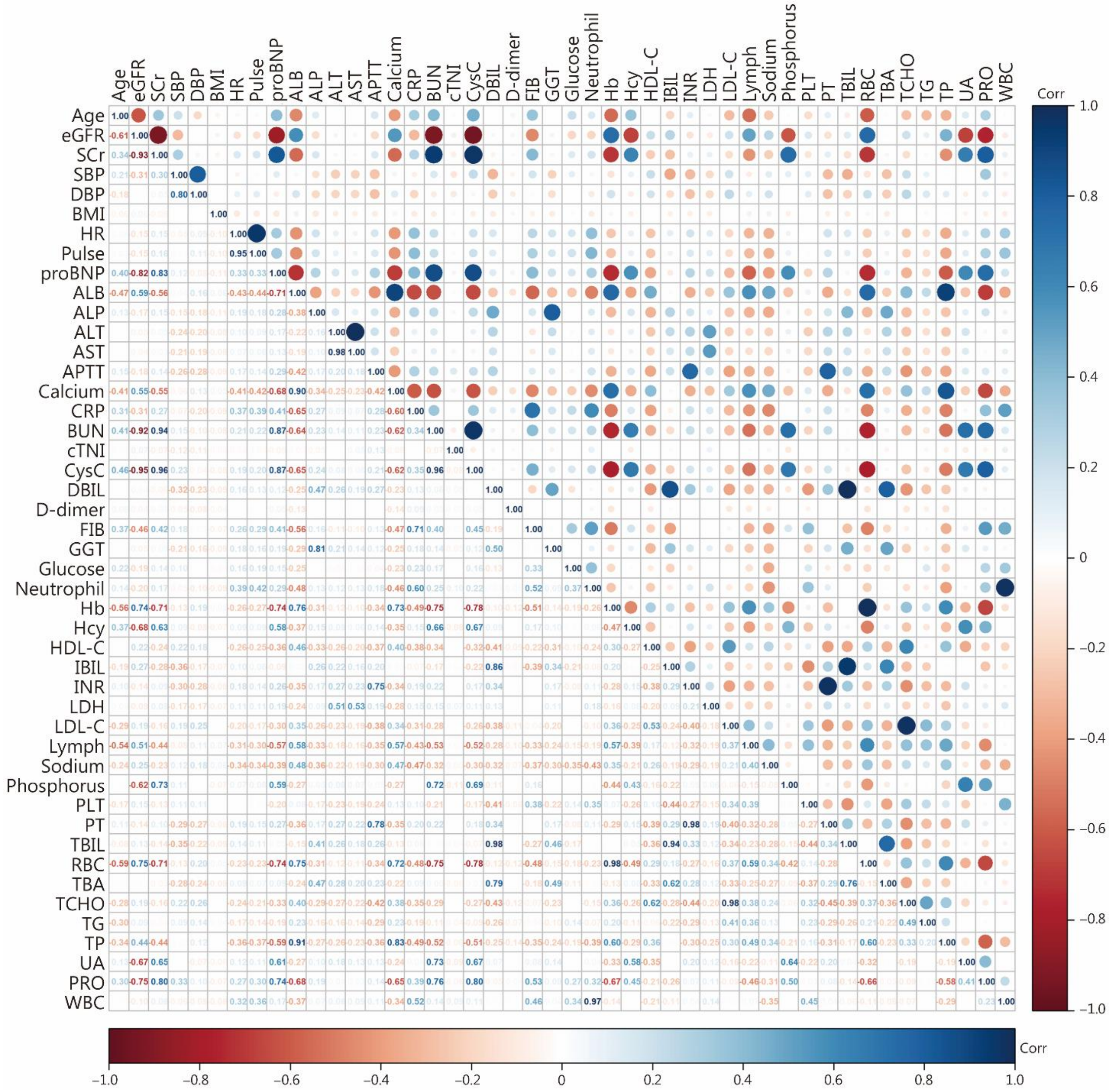

Fig. S2 Multicollinearity results for continuous variables in the derivation cohorts. eGFR estimated glomerular filtration rate, SCr serum creatinine, SBP systolic blood pressure, DBP diastolic blood pressure, BMI body mass index, HR heart rate, proBNP pro-brain natriuretic peptide, ALB albumin, ALP alkaline phosphatase, ALT alanine aminotransferase, AST aspartate aminotransferase, APTT activated partial thromboplastin time, CRP C-reactive protein, BUN blood urea nitrogen, cTNI cardiac troponin I, CyscC cystatin C, DBIL direct bilirubin, FIB fibrinogen, GGT gamma-glutamyltransferase, Hb hemoglobin, Hcy homocysteine, HDL-C high density lipoprotein cholesterol, IBIL indirect bilirubin, INR international normalized ratio, LDH lactate dehydrogenase, LDL-C low density lipoprotein cholesterol, Lymph lymphocyte, PLT platelet count, PT prothrombin time, TBIL total bilirubin, RBC red blood count, TBA total bile acid, TCHO total cholesterol, TG triglyceride, TP total protein, UA uric acid, PRO urine protein, WBC white blood count, Corr correlation coefficients

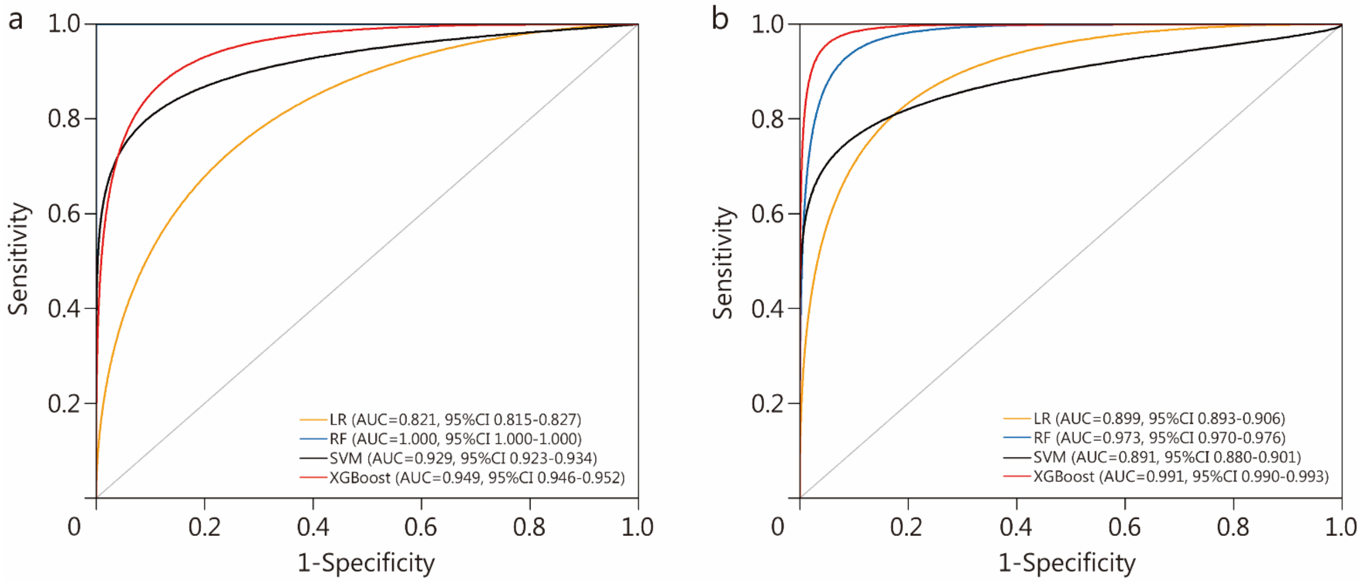

Fig. S3 The AUCs for moderate to severe AKI and AKI requiring dialysis in the derivation cohorts. **a** The AUC for moderate to severe AKI in the derivation cohort. **b** The AUC for AKI requiring dialysis in the derivation cohort. AUCs area under the receiver operating characteristic curves, AKI acute kidney injury, LR logistic regression, RF random forest, SVM supported vector machine, XGBoost eXtreme gradient boosting

References

1. Khwaja A. KDIGO clinical practice guidelines for acute kidney injury. Nephron Clin Pract. 2012;120(4):c179-84.
2. Xu X, Nie S, Zhang A, Jianhua M, Liu H-P, Xia H, et al. A New Criterion for Pediatric AKI Based on the Reference Change Value of Serum Creatinine. J Am Soc Nephrol. 2018;29(9):2432-42.
3. Bruce A, Bruce P. Regression and prediction. Practical statistics for data scientists. 1st ed. O'Reilly Media, Inc.; 2017.
4. Shapley LS. A value for n-person games. Princeton: Princeton University Press; 1953.
5. Graf E, Schmoor C, Sauerbrei W, Schumacher M. Assessment and comparison of prognostic classification schemes for survival data. Stat Med. 1999;18(17-18):2529-45.
6. Huang Y, Li W, Macheret F, Gabriel RA, Ohno-Machado L. A tutorial on calibration measurements and calibration models for clinical prediction models. J Am Med Inform Assoc. 2020;27(4):621-33.
